# Quantification of a Novel Photosensitizer of Chlorin e6-C15-Monomethyl Ester in Beagle Dog Plasma Using HPLC: Application to Pharmacokinetic Studies

**DOI:** 10.3390/molecules22050693

**Published:** 2017-04-26

**Authors:** Xiuxiu Li, Jun Wen, Jingjing Jiang, Xin Zhao, Tingting Zhou, Guorong Fan

**Affiliations:** 1 Shanghai Key Laboratory for Pharmaceutical Metabolite Research, School of Pharmacy, Second Military Medical University, Shanghai 200433, China; Lixiuxiu0217@163.com (X.L.); wenjunapple@163.com (J.W.); jiangjingj8@163.com (J.J.); 2 Department of Pharmaceutical Analysis, School of Pharmacy, Second Military Medical University, No. 325 Guohe Road, Shanghai 200433, China; 3 Department of Pharmaceutical Analysis, School of Pharmacy, China Pharmaceutical University, No. 24 Tong Jia Xiang, Nanjing 210009, China; yoyo0132@163.com; 4 Department of Clinical Pharmacy, Shanghai General Hospital, School of Medicine, Shanghai Jiaotong University, No. 100 Haining Road, Shanghai 200025, China; 5 School of Medicine, Tongji University, No. 1239 Siping Road, Shanghai 200092, China

**Keywords:** photosensitizer, chlorin e6-C15-monomethyl ester, HPLC-UV, pharmacokinetics

## Abstract

Chlorin e6-C15-monomethyl ester (CMME) is a novel photosensitizer, which is synthetized from the degradation products of silkworm excrement. Preclinical studies on the promising photosensitizer CMME are necessary to determine its therapeutic efficacy and druglikeness. A high-performance liquid chromatography with UV detection (HPLC–UV) method was established for the determination of CMME in beagle dog plasma. The sample preparation involved a protein-precipitation method with acetonitrile after the addition of tanshinone IIA as an internal standard (IS). CMME and the IS were separated on a Diamonsil C18 (2) column (100 mm × 4.6 mm, 5 μm) with a isocratic system of methanol–water containing 20 mM ammonium acetate with 0.3% glacial acetic acid (85:15, *v*/*v*). The flow rate was 1.0 mL/min with UV detection using a wavelength of 400 nm. The method was sensitive enough with a lower limit of quantitation (LLOQ) of 0.05 μg/mL and had a good linearity (*r*^2^ > 0.999) over the linear range of 0.05–5.00 μg/mL. The intra-day and inter-day accuracies ranged from 98.5% to 102.8% and precisions (RSD) were within 6.8%. The validated method was successfully applied to the pharmacokinetic study of CMME after intravenous administration of single and multiple doses in beagle dogs.

## 1. Introduction

Photodynamic therapy (PDT) is a noninvasive method used for the treatment of malignant tumors based on the combination of the photosensitizer (PS) and light irradiation [1]. It can be used either as a primary or adjunctive treatment for solid cancers of various parts of the body including the bladder, esophagus, head and neck, brain, lung, prostate, intraperitoneal cavity, breast, prostate and skin [2]. Recently, PDT had gained more and more attention due to its significant advantages of destroying tumor cells effectively and selectively without injuring surrounding healthy tissues, and had been widely used as a new treatment that improves the quality of life of patients [2,3,4,5,6].

**Figure 1:**
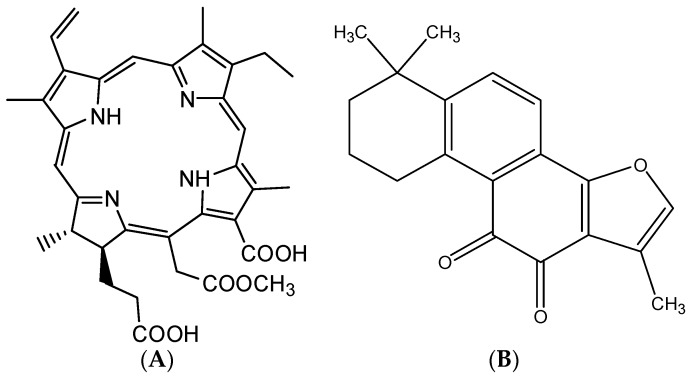
Chemical structures of (**A**) CMME; (**B**) Tanshinone IIA (IS).

In PDT the PS allows for the transfer and translation of light energy into a type II chemical reaction, producing singlet oxygen (^1^O_2_) by transferring energy to the surrounding ground-state oxygen, which was extremely cytotoxic and was believed to play a major role in cell killing [2,7,8,9]. Chlorin e6, the most studied and best known photosensitizer used in PDT in many countries worldwide, possesses clear structure, better tumor targeting ability, strong photodynamic activity and lower cytotoxicity [3,10,11]. 2,7,12,18-Tetramethyl-3-ethenyl-8-ethyl-13-carboxyl-15-formyloxyethyl-17-propionyloxy-17, 18-chlorin (CMME, Figure 1A) is a derivative of chlorin e6, which is a new second generation drug development candidate for tumor photodynamic therapy [12]. The absorption coefficient of CMME is approximately one order of magnitude higher than that of the current PDT drugs such as sodium porfimer and xibofen, since the photosensitive action spectrum of CMME is in the best red light band [13,14]. Besides the mentioned benefits, CMME also has the advantage of chemical stability, a high tumor/normal tissue distribution ratio, and strong photosensitizing and antineoplastic activity [15,16].

Due to its various advantages described above, further research and development of CMME is of great significance. The pharmacokinetic properties of a drug play an important role in its therapeutic safety and clinical rational drug use. Yang et al. reported a FLD method for determination of CMME in Sprague-Dawley rat plasma with the LLOQ of 0.5 μg/mL, which was higher than that in this work [17]. However, the fluorescence efficiency was easily affected by temperature, solvent and acidity, which affected the shape and intensity of the fluorescence spectrum [17,18]. Thus, a suitable liquid chromatographic method that will allow analysis of CMME in pre-clinical is still lacking.

CMME, is a large heterocyclic aromatic molecule with a conjugated π-system, consisting of a core of three pyrrole rings and one reduced pyrrole ring coupled through four methine linkages [15]. Owing to the strong conjugated system, CMME has strong ultraviolet absorption and a UV method should be sensitive enough for direct analysis [15,19]. In addition, because of the simple HPLC conditions and straightforward sample pre-treatment procedure, the method is easy and fast to perform [20]. However, up to date, there are no reports on the determination of CMME using HPLC-UV.

The purpose of this study was to develop and validate a HPLC method for quantitative determination of CMME in beagle dog plasma. The method was evaluated in terms of selectivity, sensitivity, linearity, accuracy, precision and stability in accordance to the recommendations published by the FDA, and it was successfully applied to quantitative levels of CMME in beagle dog pharmacokinetic studies.

## 2. Results and Discussion

### 2.1. Method Development

#### 2.1.1. Optimization of Chromatographic Conditions

An appropriate wavelength is important for good sensitivity. CMME has strong UV absorptions at the wavelengths of 400 nm, 498 nm and 660 nm due to its special conjugated structure, which was consistent with the literature [21]. The detection wavelength was set at 400 nm due to the stronger absorption compared with 498 and 660 nm.

Analysis was performed on several HPLC columns, including a Welch Ultimate Polar-RP column (150 × 4.6 mm, 5 μm), a Diamonsil C18 column (150 × 4.6 mm, 5 μm) and a Diamonsil C18 (2) column (100 × 4.6 mm, 5 μm). After careful comparison of these columns, a Diamonsil C18 (100 mm × 4.6 mm, 5 μm) was finally selected to achieve an efficient chromatographic as it showed better separation and peak shape. The chromatographic conditions, especially the composition of the mobile phase, were optimized through several trials to achieve good resolution and symmetric peak shapes for each analyte and the IS, as well as a short run time. When methanol-water or acetonitrile-water systems was used, there was no retention of CMME. In order to enhance the retention of CMME, buffer salts were considered to be added to the mobile phase. At the beginning, different concentration of the PBS buffer was selected for the optimization studies, since CMME dissolve in 60% PBS buffer and 40% methanol. Although the appropriate retention time and better resolution were achieved, the column pressure could be increased by increasing of the buffer concentration. When samples were analyzed continuously, the column pressure was increased even if the lowest concentration of PBS buffer was added, which was detrimental to the continuous analysis.

Secondly, with ammonium acetate buffer instead of phosphate buffer, retention time and peak shape are also acceptable, but the response is not very high. The addition of acetic acid to the mobile phase could improve the response, resolution and peak shape of CMME and IS. Different acetic acid concentrations were tested to choose the optimal mobile phase. It was found that the percentage of the acetic acid in the mobile phase had great influence on the retention time of CMME, while did not affect the IS. Thus 0.3% acetic acid in the mobile phase appeared to be optimal combination to separate CMME and the IS with better sensitivity. In addition, as the concentration of organic phase increased, the retention time of the analyte was shorter and the resolution was worse. When the organic phase concentration decreased, the analysis took longer. In this work, the use of an (organic solvent:water, 85:15; *v*/*v*) mixture as mobile phase gave better resolution and running time. To summarize, the best results were achieved with the Diamonsil C_18_ (2) column (100 mm × 4.6 mm, 5 μm) column, flushed with a combination of methanol and acidified ammonium acetate buffer (85:15, *v*/*v*) as the mobile phase, pumped at a flow rate of 1.0 mL/min at 25 °C.

#### 2.1.2. Optimization of the Sample Preparation

In order to improve the detection sensitivity and increase the extraction recovery, sample pretreatment methods were investigated. The extraction of plasma samples was optimized in our preliminary studies by comparing liquid–liquid extraction, solid-phase extraction and protein precipitation (Table 1). The results showed that the exaction efficiency of all three sample pretreatment methods was acceptable and had no significant difference. However, as a novel photosensitizer with a chlorin e6 molecular skeleton, CMME is not very stable and easily degraded under light conditions [22]. Fast operation under dark conditions was thus necessary owing to its optical instability. Therefore, neither liquid-liquid extraction nor solid-phase extraction was suitable for CMME extraction from plasma because of their tedious time-consuming and wasteful nature [23]. Protein precipitation was more advisable and advantageous in the present work because of the simple and fast performance. Two protein precipitation solvents—methanol and acetonitrile—were tried at volume ratios of 3:1 and 2:1, respectively. Using 2:1 acetonitrile as the protein–precipitating agent gave higher sensitivity and satisfactory extraction efficiency, hence acetonitrile at volume ratio of 2:1 was used as the protein–precipitating agent.

#### 2.1.3. Selection of the Internal Standard

It is necessary to screen a suitable internal standard (IS) to track a target compound in any in vivo quantitative study. According to the structure, polarity and chromatography behavior of CMME, as well as a strong absorption at 400 nm, four compounds with relatively strong wavelength absorption including sodium tanshinone IIA sulfonate, tanshinone IIA, salvianolic acid B and ligustilide were selected as IS candidates for. Sodium tanshinone IIA sulfonate and salvianolic acid B were abandoned because were not retained under the optimized chromatographic conditions. Ligustilide was also excluded due to its poor stability and interference by endogenous matrix. On the other hand, tanshinone IIA (Figure 1B) had an appropriate retention time and sensitivity, therefore, it was finally selected as the internal standard.

### 2.2. Method Validation

#### 2.2.1. Specificity

**Figure 2:**
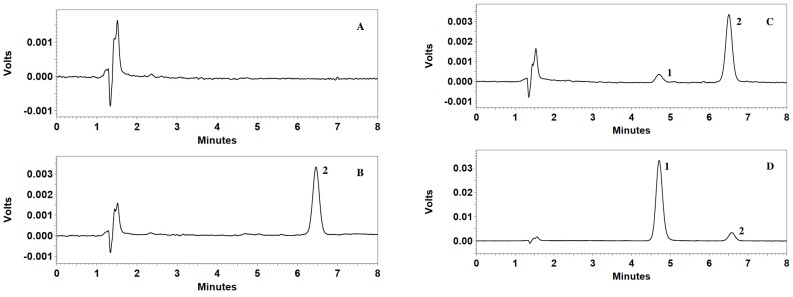
Typical chromatograms of HPLC of the blank dog plasma (**A**); blank dog plasma spiked with IS (25.00 μg/mL) (**B**); blank dog plasma spiked with CMME (0.05 μg/mL) and IS (25.00 μg/mL) (**C**) and dog plasma sample obtained 2 min after a single intravenous administration of 1.2 mg/kg spiked with IS (**D**). Peak 1: CMME; peak 2:IS.

A good separation was achieved and no significant endogenous interferences with the analyte or the IS were observed. Representative chromatograms of the blank plasma, blank plasma spiked with IS, blank plasma spiked with CMME at LLOQ and plasma samples obtained from pharmacokinetic studies are shown in Figure 2.

#### 2.2.2. Linearity and Lower Limit of Quantification

The calibration curve of CMME was linear over the concentration range of 0.05–5.00 μg/mL for beagle dog plasma. The representative linear equation was *Y* = 2.4696*X* + 0.0034 (*r*² = 0.9991) for the dog plasma. *Y*, peak area ratio (CMME/IS) and *X*, added CMME concentration (μg/mL). The LLOQ for dog plasma was proved to be 0.05 μg/mL with a precision of 5.9%, which was sufficient for pharmacokinetic study. Unknown sample concentrations exceeding the range were diluted appropriately with control blank plasma and re-assayed

#### 2.2.3. Precision and Accuracy

As shown in Table 2, the method was reproducible and accurate for the determination of CMME in dog plasma. The intraday precision was less than 5.9%, and the accuracy was in the range of −1.5% to 2.8%, whereas the interday precision was less than 6.8%, and the accuracy was between 0.9% and 2.6% for each QC level of CMME

#### 2.2.4. Recovery

Average extraction recovery of CMME and IS in dog plasma were greater than 90.3% (Table 3). These results indicated that there was high recovery from dog plasma.

#### 2.2.5. Stability

As was illustrated in Table 4, CMME was found to be stable in the dog plasma under various storage conditions with acceptable accuracy (from −3.3% to 6.9%) and precision (between 1.0% and 6.1%). The data demonstrated that CMME was stable in dog plasma after three freeze-thaw cycles, for 30 days under −80 °C, and for at least 2 h at the room temperature, a period of 24 h needed to process a batch of samples in the autosampler tray.

#### 2.2.6. Sample Dilution

The accuracy expressed as RE% and precision expressed as RSD% of these samples were shown in Table 5. The results demonstrated that diluting high concentration samples with blank plasma would not affect the accuracy and precision of the assay.

### 2.3. Application to Pharmacokinetic Studies

As far as we know, there have been no reports of pharmacokinetic studies of CMME in beagle dog plasma. The validated HPLC method was therefore successfully applied to evaluate the pharmacokinetic study of CMME in dog plasma.

**Figure 3:**
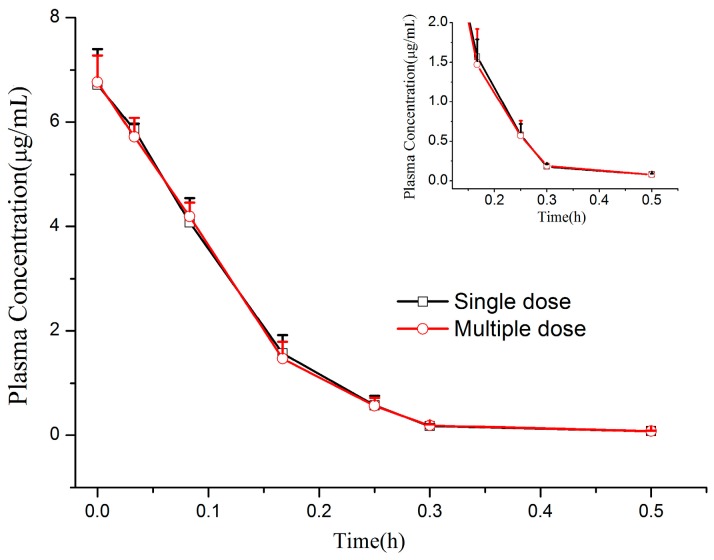
Mean plasma concentration–time profiles of CMME after single and multiple intravenous administrations (1.2 mg/kg/day, three times a day for seven days).

Non-compartmental mode was used to calculate the pharmacokinetics parameters with BAPP, Version 3.0 (Center of Drug Metabolism and Pharmacokinetics, China Pharmaceutical University, Nanjing, China). The mean plasma concentration vs. time profiles of CMME after single and multiple doses (1.2 mg/kg) administration in beagle dogs are shown in Figure 3 and a comparison of the main pharmacokinetic parameters of CMME is presented in Table 6. It was shown that the plasma concentration of CMME decreased sharply and was reduced to its lowest level 0.5 h after its intravenous administration at 1.2 mg/kg. The apparent elimination half-life (t_1/2_) was 0.08 ± 0.01 h, indicating CMME could be cleared quickly from the dog plasma. Half-life of CMME was very short, indicating that the elimination was very fast and it was difficult to maintain a stable plasma concentration, which had a certain guiding significance for clinical dosing regimens. Thus, in clinical application it would be better for CMME to be administrated in a higher dose and with a longer interval between dosing or by intravenous infusion.

A multi-dose test dosing regimen was routinely administered three times a day (every 8 h) in order to observe the exposure and accumulation of CMME in vivo, which was significant for the safety interpretation. After multiple dosing of 1.2 mg/kg/day, all the trough concentrations were below the lower limit of quantitation. The accumulation ratios of AUC_0–τ_ and C_0_ of CMME were 0.99 and 1.01, based on day 7 to day 1. The analysis of variance of the pharmacokinetic parameters and plasma concentrations between day 1 and day 7 showed no difference. These results indicated that there was no evidence of drug accumulation in the plasma during multiple-dosing at 1.2 mg/kg/day for seven consecutive days in dog.

### 2.4. Comparative Analysis of CMME by Using Fluorescence Spectrophotometry and HPLC-UV

The use of fluorescence spectrophotometry for the quantification of CMME has been published [17], however no detailed pharmacokinetic parameters, nor any analysis of the pharmacokinetic behavior were given in the previous article. Since the conjugate structure of CMME contributes to its strong UV absorption, it can be detected directly with HPLC-UV. In this study, the pharmacokinetics were investigated in detail and demonstrated that the result was similar to the previous research of no accumulation for CMME. Compared with the reported results, our method for the determination of CMME described here represented a rapid, sensitive, and efficient analytical method. The sensitivity of this method was 10 times higher than that of the previous method with the LLOQ from 0.5 μg/mL down to 0.05 μg/mL.

## 3. Experimental Section

### 3.1. Chemicals and Reagents

A CMME reference substance (Lot: 20150112) and powder injection (98.73% purity) were synthesized at Haining Green Liter Pharmaceutical Technology Co., Ltd. (Haining, China). Tanshinone IIA (internal standard, IS, 98% purity) was purchased from the Dalian Meilun Biotechnology Co. Ltd. (Dalian, China). Methanol and glacial acetic acid, both of HPLC grade, were purchased from Tedia (Fairfield, CA, USA). Ammonium acetate (analytical reagent grade) was purchased from Shanghai Chemical Reagent Company (Shanghai, China). Ultra-pure water was prepared in-house using a Milli-Q system from Millipore (Bedford, MA, USA).

### 3.2. Instruments

The analysis was performed on a Shimadzu LC-10 series HPLC system (Shimadzu, Kyoto, Japan), including two LC-10A pumps, a SIL-10ADvp autosampler, a CTO-10ASvp column oven and a SPD-10A UV detector. The data was collected and processed using Shimadzu CLASS-VP software (Version 6.14 SP1, Shimadzu, Kyoto, Japan).

### 3.3. Chromatographic Conditions

Chromatographic separation was performed on a Diamonsil C18 column (100 mm × 4.6 mm, 5 μm; Dikma, Beijing, China). The mobile phase consisted of methanol and 20 mM ammonium acetate (containing 0.3% glacial acetic acid) (85:15, *v*/*v*) was delivered at a flow rate of 1.0 mL/min. The column temperature was 25 °C with UV detection at 400 nm. This method was applied to analyze the plasma samples from beagle dogs dosed with CMME.

### 3.4. Preparation of Calibration Standards and Quality Control Samples

The stock solutions of CMME were prepared at concentrations of 1.00 mg/mL in 60% PBS buffer and 40% methanol. A series of standard working solutions were prepared by further dilution of the stock solutions with the diluents (methanol–water: 80:20, *v*/*v*). The stock solutions of IS were prepared at concentrations of 0.50 mg/mL in methanol. The IS working solution was diluted with methanol–water (80:20, *v*/*v*) to get a final concentration of 250.00 μg/mL. All working solutions were stored at +4 °C prior to sample processing. Calibration standards of CMME in the dog plasma were prepared at concentrations of 0.05, 0.10, 0.20, 0.50, 1.00, 2.00 and 5.00 μg/mL along with quality control (QC) standards at concentrations of 0.15 μg/mL (LQC, low QC), 0.80 μg/mL (MQC, middle QC), and 4.00 μg/mL (HQC, high QC). The concentration of the IS working solution was 250.00 μg/mL.

### 3.5. Sample Preparation

The samples of beagle dog plasma were taken out from −80 °C freezer and thawed at room temperature prior to use. The samples were vortexed adequately before processing. An aliquot of 100 μL plasma sample and 10 μL IS working solution were mixed together by vortexing for 30 s and then extracted by protein precipitation with 200 μL acetonitrile for 3 min by a vortex mixer. After centrifugation at 12,000 rpm for 10 min, the supernatant was transferred into vial and a 20 μL aliquot was injected into the HPLC system for analysis.

### 3.6. Method Validation

The bioanalytical method was fully validated according to the FDA guidelines [24].

#### 3.6.1. Specificity

Specificity of the method was investigated by blank plasma samples from six different sources, blank plasma spiked with IS, blank plasma spiked with CMME at LLOQ and plasma samples in a preclinical study. The method is selective if the response of the interfering peaks at the retention time of the drug is less than 20% of the mean response of the six extracted samples at LLOQ.

#### 3.6.2. Linearity and Lower Limit of Quantitation

The linearity was assessed by plotting the peak area of the CMME/IS ration against the concentration of CMME (0.05–5.00 μg/mL) on three consecutive days with least-squares linear regression analysis. Standards should not deviate by more than 15% of nominal concentrations, except at LLOQ where the standard should not deviate by more than 20%.The acceptance criterion for the standard curve is that at least 75% of non-zero standards should meet the above criteria, including the LLOQ. The LLOQ was established using five samples independent of the standards and was determined with a precision less than 20% and accuracy within ±20%.

#### 3.6.3. Recovery

The extract recovery was evaluated by comparing the mean peak areas for extracted samples (*n* = 5) at three concentrations (0.15, 0.80 and 4.00 μg/mL) with the mean peak areas of unextracted standards that represent 100% recovery. The recovery of IS was also evaluated in the same way.

#### 3.6.4. Precision and Accuracy

The intra-day and inter-day precision and accuracy were assessed by analyzing five replicates of four concentration levels of QC samples (LLOQ, LQC, MQC, and HQC) on the same day and on three validation days. The criteria for acceptability of the data included accuracy within ±15% (20% for LLOQ) relative error (R.E.) from the nominal values and a precision within 15% (20% for LLOQ) relative standard deviation (R.S.D.).

#### 3.6.5. Stability

Stability studies were assessed by analyzing three replicates of the QC samples at two concentrations. The long-term stability was assessed after storage of the samples for three months at −80 °C The freeze and thaw stability was evaluated after three freeze (−80 °C) and thaw cycles. The short-term stability was evaluated after exposure of the samples to room temperature for 1 and 2 h. The post-preparative stability was analyzed after storage of the samples in the autosampler for 12 h and 24 h at room temperature. Stability sample results should be within 15% of nominal concentrations.

#### 3.6.6. Sample Dilution

To demonstrate the ability of diluting plasma samples containing CMME at concentrations above the assay upper limit of calibration curve, a set of plasma samples were prepared containing CMME at a concentration of 8.00 μg/mL, 20.00 μg/mL, 40.00 μg/mL and stored at −80 °C overnight prior to analysis. After thawing at room temperature, the spiked samples were diluted with blank plasma to generate the final concentration of 4.00 μg/mL. Then the samples (*n* = 5) were processed and analyzed. The accuracy expressed as RE% and precision expressed as RSD% of these samples should be within ±15%.

### 3.7. Application of the Method in Pharmacokinetic Study

Beagle dogs, weighing 10.2 ± 0.8 kg, half male and half female, were obtained from Experimental Animal Center of Second Military Medical University (Shanghai, China; animal permit number: SCXK (Hu) 2012-0003). The animals were fasted overnight with free access to water for at least 12 h before administration. The CMME powder was dissolved in 0.9% saline solution with the concentration of 4 mg/mL. Twelve beagle dogs were divided into two groups and administered by left forelimb intravenous injection at a single dose of 1.2 mg/kg and multiple doses of 1.2 mg/kg/day for seven consecutive days (three times a day). Beagle dog blood samples (approximately 0.5 mL) were collected in heparin-coated tubes prior before administration and at different time points (0, 0.033, 0.083, 0.167, 0.25, 0.5, 1, 2, 3 and 4 h) after single-dose intravenous administration. For the multiple-dose studies, the blood-sampling scheme mentioned above was applied to beagle dogs on days 1 and 7, while blood samples were only collected immediately prior to every dosage on days 4–6 for the determination of trough concentrations. All blood samples were centrifuged at 6500 rpm for 10 min. The plasma was separated and frozen below −80 °C until assay.

At the time of measurement, first three time points (i.e., 0, 0.033 and 0.083 h) of single-dose and multiple-dose plasma samples were diluted with blank plasma of a dilution factor of 5 (to 80 μL of blank plasma, added 20 μL of i.v. treated dog plasma sample), because these points were out of our validation range. As for the other time points, the measured values were not diluted. The pharmacokinetic parameters of CMME were calculated using the non-compartmental model with the BAPP 3.0 software (Center of Drug Metabolism and Pharmacokinetics, China Pharmaceutical University, Nanjing, China).

## 4. Conclusions

A specific, sensitive, accurate and rapid HPLC method for determination of CMME in dog plasma was developed and validated for the first time. It was successfully applied in the determination of the pharmacokinetic properties of CMME. We characterized the in vivo pharmacokinetics in dogs after intravenous administration of single and multiple doses. The method was sensitive and robust and could be used in further pharmacokinetic studies and in routine analysis of CMME as a PDT sensitizing agent. These results provide vital guidance for further preclinical research and the subsequent clinical trials.

## Figures and Tables

**Table 1:** Extraction recovery using different sample pretreatment methods of PPT, LLE and SPE.

Pretreatment Method	Pretreatment Solvent	Recovery (%)	
		CMME	IS
PPT	Methanol (1:2)	96.6 ± 1.7	91.3 ± 2.5
	Methanol (1:3)	96.5 ± 1.6	92.7 ± 3.9
	Acetonitrile (1:2)	98.9 ± 0.8	99.0 ± 1.6
	Acetonitrile (1:3)	97.8 ± 1.4	97.6 ± 1.4
LLE	Ethyl acetate	-	-
	Ethyl acetate and hydrochloric acid	97.7 ± 1.6	82.9 ± 2.4
SPE	Methanol	81.5 ± 1.2	85.8 ± 2.1

“-” indicates that the extraction recovery was very low and did not provide acceptable data.

**Table 2:** Intraday and interday precision (% RSD) and accuracy (% RE) of the assay for CMME in dog plasma.

Added Con.	Found Con.	RSD (%)	RE (%)
(μg/mL)	(Mean ± SD)		
Intraday (*n* = 5)			
0.05	0.05020 ± 0.00	5.9	−1.5
0.15	0.1517 ± 0.00	2.2	−0.7
0.80	0.8173 ± 0.02	2.8	0.4
4.00	4.187 ± 0.12	3.0	2.8
Interday (*n* = 15)			
0.05	0.05220 ± 0.00	6.8	2.5
0.15	0.1566 ± 0.01	3.4	2.6
0.80	0.8219 ± 0.02	1.9	0.9
4.00	4.148 ± 0.12	3.0	1.9

**Table 3:** Recovery of CMME and IS in dog plasma (*n* = 5).

Sample	Added Con.	Recovery (%)
	(μg/mL)	(Mean ± SD)
LQC	0.15	91.1 ± 2.2
MQC	0.80	90.5 ± 1.8
HQC	4.00	90.3 ± 2.3
IS	25.00	96.6 ± 1.8

**Table 4:** Stability of CMME in dog plasma under various storage conditions (*n* = 3).

Sample Condition	Added Con. (μg/mL)	Found Con. (Mean ± SD)	RSD (%)	RE (%)
Three freeze–thaw				
cycles at −80 °C				
	0.15	0.1576 ± 0.01	6.1	2.4
	4.00	3.970 ± 0.04	1.0	−3.3
Auto-sampler stability, 24 h				
	0.15	0.1557 ± 0.01	5.1	1.2
	4.00	4.028 ± 0.06	1.4	−1.9
At room temperature, 2 h				
	0.15	0.1584 ± 0.01	4.3	2.9
	4.00	4.027 ± 0.08	1.9	−1.9
storage stability (30 Day)				
	0.15	0.1645 ± 0.01	3.9	6.9
	4.00	4.003 ± 0.13	3.3	−2.5

**Table 5:** Sample dilution accuracy and precision of the assay for CMME in Beagle dog plasma (*n* = 5).

Dilution Factor	Added Con. (μg/mL)	Found Con. (Mean ± SD)	RSD (%)	RE (%)
2	8.00	8.265 ± 0.17	2.0	1.5
5	20.00	20.38 ± 0.30	1.5	0.1
10	40.00	40.90 ± 0.75	1.8	0.5

**Table 6:** Mean pharmacokinetic parameters of CMME after i.v. of a single-dose of 1.2 mg/kg and multiple-dose of 1.2 mg/kg/day (three times a day) for seven days to beagle dogs (mean ± SD) (*n* = 6).

Parameters	1.2 mg/kg	1.2 mg/kg (day 7)
AUC_0–τ_ (μg∙h/mL)	0.83 ± 0.06	0.82 ± 0.04
t_1/2_ (h)	0.08 ± 0.01	0.08 ± 0.01
CL (L/kg/h)	1.44 ± 0.10	1.44 ± 0.07
MRT (h)	0.10 ± 0.01	0.09 ± 0.01
C_0_ (μg/mL)	6.72 ± 0.63	6.77 ± 0.51

## Abbreviations

CMME	chlorin e6-C15-monomethyl ester
HPLC	high performance liquid chromatography
UV	ultraviolet
IS	internal standard
LLOQ	lower Limit of quantitation
RSD	relative standard deviation
PDT	photodynamic therapy
PS	photosensitizer
^1^O_2_	singlet oxygen
SD	standard deviation
FDA	USA Food and Drug Administration
PBS	phosphate buffered saline
PPT	protein precipitation
LLE	liquid-liquid extraction
SPE	solid phase extraction
RE	relative error
QC	quality control
LQC	lower concentration quality control
MQC	medium concentration quality control
HQC	high concentration quality control
AUC	area under the curve
t_1/2_	half-life time
MRT	mean residence time
CL	clearance
C_max_	peak concentration
t_max_	time to reach C_max_
